# 

**Published:** 1887-12

**Authors:** 


### INDEX TO VOLUME LV.

A

PAGE

Abdomen, Penetrating Gunshot Wound of the, E. Andrews 125
Abdomen, Gunshot Wound of, with Perforation of the Intestines, F. T. Andrews 127
Abortion, H. Carson 94
Abstracts and Extracts 142–183, 422–424
Albumen in the Urine, and Its Tests 151
Alexander's Operation, Round Ligaments from a case of, H. T. Byford 105
Andrews, Edmund, The Cure of Fistula in Ano without Operation 46
Penetrating Gunshot Wound of the Abdomen 125
Angina Pectoris without Lesion of the Coronary Arteries 156
Antisepsis in Abdominal Operations—Fenger and Holmes 1
Atheroma of the Left Coronary Artery, resulting in Aneurism of the Apex of the Left Ventricle, R. Tilley 285

B

Babcock, R. H., A case of Peri-endocarditis 116
Bacteria and Tetanus 140
Belfield, W. T., A case of Mercurial Necrosis, with Specimen 120
Billings, F. S., The Necessity of a Uniform Standard of Education in all American Medical Schools 83
Blandford, B. Fielding, Treatment of Recent Cases of Insanity in Asylums and Private Houses 381
Bombaugh, C. C., The Later Phases of Medical Examinations for Life Insurance 189
Bone, Grafting of Human 169
Book Notices and Reviews 123, 184, 366, 427
Books Received 284, 427, 428
Brown, J. Y., Transverse Fracture of the Patella 131
Burns, A New Remedy for 183
Byford, H. T., Curettes 105
Round Ligaments, from a Case of Alexander's Operation 105

C

PAGE

Caldwell, Charles, Enlarged Thyroid a Cause of Transverse Presentation 109
Cancer of the Testicle, with Secondary Pleurisy, J. A. Robison 114
Carson, Hiram, Abortion 94
Causes of Subcutaneous Inflammation and Suppuration 145
Cell Antagonism, E. H. Gregory 74
Chicago Gynæcological Society, Proceedings of the April Meeting 105
Chicago Medical Society, Proceedings of Stated Meeting of June 20 113
Proceedings of Stated Meeting of July 5 118
Chorea of Early Childhood, The Rheumatic Element in 165
Cocaine, The Therapeutic Uses of 167
Concerning Myœdema, Virchow, Translator E. Wing 30
Constipation, Obstinate case of 142
Co-relation of General and Dental Medicine and Surgery, G. F. Lydston 88
Correspondence, Foreign, London Letter 135
Chiavari Letter 137, 410—418
Bermuda Letter 425
Cremation, Report of Committee 99
Curettes, H. T. Byford 105
Curtis, Lester, Paralytic Phenomena in Simple Psychosis, Translation 143

D

Davis, N. S., Jr., Chronic Meningitis 49
Diphtheria, H. L. Getz 100
Dyspepsia in Infants 142
Dyspeptic Origin of Visual Troubles 156

E

Earl, C. W., Cystic Sarcoma of the Right Ovary 106
Editorial, Ninth International Medical Congress 139, 195
Tyrotoxicon in Food 139
Bacteria and Tetanus 140
Twelfth Biennial Meeting of the Italian Medical Association 380

F

PAGE
Fallopian Tubes, Morbid Anatomy of the 171
Fenger, Christian, Antisepis in Abdominal Operations 1
Proliferating Cystoma, with Partial Intraligamentous Development 108
Vaginal Hysterectomy 367
Fistula in Ano, The Cure of, without Operation, Andrews 46
Flint, Austin, Sr., Portrait of 11
French, G. F., The Chief Source of Danger in the use of the Uterine Sound 93

G

Galvanic Current in Gynæcology, The Value of the, Martin 34
Garnett, A. Y. P., Surgical Treatment of Suppurative Pleuritis in Children 96
Getz, H. L., Diphtheria 100
Gonorrhœal Peritonitis, J. E. Green 122
Gunshot Wound of the Abdomen, with Perforation of the Intestines, F. T. Andrews 127
Gunshot Wound of the Abdomen, Penetrating, E. Andrews 125
Grafting of Human Bone 169
Green, J. E., Gonorrhœal Peritonitis 128
Gregory, E. H., Cell Antagonism 74

H

Haven, Joseph, Mechanical Support for Maintaining the Lithotomy Position 121
Holmes, Bayard, Antisepsis in Abdominal Operations 1
Hyoscine, The Action of Hydrochlorate of 170
Hysterectomy, Vaginal, Christian Fenger 367

I

Illinois, The Amended Medical Practice Act of 53
Proceedings of last Quarterly Meeting of State Board of Health of 56
Inebriety, Lack of Interest by the General Profession, in the Disease of, Domestic Correspondence 62
Intra-Pulmonary Medication, A New Method of, G. W. McCaskey 101
Intra-Peritoneal Injuries, The Treatment of 160
Intestinal Obstruction from Impaction of a Gall-Stone in the Ileum 174
International Medical Congress, The 196, 284, 288, 366
Insane Pauper Immigration, Editorial 61
Insanity, Treatment of Recent Cases of, in Asylums and in Private Houses, B. Fielding Blandford 381

L

PAGE
Laparotomy for Visceral Injuries, McGraw 5
Lens, Observations on Displacement of the Crystalline, from Congenital and other causes, J. L. Thompson 85
Life Insurance, The Later Phases of Medical Examinations for, C. C. Bombaugh 189
Lithotomy Position, Mechanical Support for Maintaining the, Joseph Haven 121
Locomotion, Interference with as a Uterine Symptom 162
Lydston, G. F., The Co-relation of General and Dental Medicine and Surgery 88

M

Mammæ, Scirrhus of the, W. C. Wile 80
Martin, F. H., The Value of the Galvanic Current in Gynæcology 34
McGraw, T. A., Laporotomy for Visceral Injuries 5
McCaskey, G. W., A New Method of Intra-Pulmonary Medication 101
Medical Association, The Meeting of the Twelfth Italian 410–421
Medical Congress, Ninth International; see also "N" 139
Mercurial Necrosis, A Case of, with Specimen, W. T. Belfield 120
Mercurialism in Lying-in Women Undergoing Sublimate Irrigation 166
Medical Congress, The International, Editorial 58
Medical Practice Act of Illinois, the Amended 53
Meningitis, Chronic, Davis Jr. 49
Mineral Waters, The Abuse of 147
Miscellany, Metaphysical Healing, A New Remedy for Burns, Deaths among the German Philosophers 64
Mississippi Valley Medical Association, Editorial 60
Mississippi Valley Medical Association 141
Morris, Placenta Praevia 84
Myxœdema, Concerning, Virchow 30

N.

Ninth International Medical Congress, Abstract of the Proceedings 196–284, 288–366
General Sessions 196, 197, 198, 232, 288, 322
Opening of the Congress and Officers Elected 196, 197
Report of the Secretary-General 197
Address by the Secretary of State 198
Address by the President of the Congress 198
Address on Bacteriology and its Therapeutic Relations, by Professor Mariano Semmola, Naples, Italy 288

PAGE

Address on the Relation of Dermatology to General Medicine, by Dr. P. G. Unna, Hamburgh, Germany ... 322
Address on the Treatment of Recent Cases of Insanity in Asylums and in Private Houses, B. Fielding Blandford ... 381
Ninth International, etc. (con.) ... 2
Section on General Medicine, 202, 259, 289, 323, 382
Section on General Surgery ... 204, 250, 313, 326, 383
Section on Military and Naval Surgery and Medicine ... 211, 244, 277, 314, 328, 386
Section on Obstetrics ... 213, 262, 309, 332, 387
Section on Gynæcology ... 206, 251, 304, 334, 391
Section on Therapeutics and Materia Medica ... 223, 238, 292, 337, 392
Section on Anatomy ... 198, 233, 293, 340, 393
Section on Pathology ... 221, 264, 316, 341
Section on Diseases of Children 226, 240, 317, 342, 394
Section on Ophthalmology 229, 254, 265, 294, 343, 396
Section on Otology ... 216, 267–297, 348, 400
Ninth International Medical, etc. (con.) ... 3
Section on Laryngology ... 224, 246, 280, 318, 354, 402
Section on Dermatology and Syphilography ... 199, 257, 299, 357, 403
Section on Public and International Hygiene ... 232, 256, 319, 360
Section on Climatology and Demography ... 210, 236, 301, 360, 406
Section on Psychological Medicine and Nervous Diseases ... 221-248, 319-361
Section on Dental and Oral Surgery. 228–235, 302, 365, 407
Section on Physiology ... 225, 264
Final Session—Adjournment ... 408

O.

Ovary, Cystic-sarcoma of the Right, C. W. Earle ... 106

P.

Pamphlets Received ... 284
Paralytic Phenomena in Simple Psychosis, Translation by Lester Curtis ... 143
Patella, Transverse Fracture of the, J. Y. Brown ... 131
Pauper, Immigration, Insane, Editorial ... 61
Perineal, Urethrotomy, External, H. O. Walker ... 94
Peritonitis, Gonorrhœal, J. E. Green ... 128

PAGE

Pericarditis and Endocarditis, a Case of, R. H. Babcock ... 116
Phthisis, Climate as a Therapeutic Agent in. 178
Placenta Prævia, Morris ... 84
Pleuritis in Children, Surgical Treatment of Suppurative, A. Y. P. Garnett ... 96
Progress in Gynæcology, O. B. Will ... 22
Proceedings of last Quarterly Meeting of State Board of Health of Illinois ... 56
Proliferating Cystoma, with Partial Intraligamentous Development, Christian Fenger 108
Pupil in its Semeiological Aspects ... 148

R.

Robison, J. A., Cancer of the Testicle, with Secondary Pleurisy ... 114
Round Ligaments from a Case of Alexander's Operation, H. T. Byford ... 105

S.

Standard of Education in all American Medical Schools, the Necessity of a Uniform, F. S. Billings ... 83
Sublimate Irrigation, Mercurialism in Lying-in Women Undergoing ... 166
Subcutaneous Inflammation and Suppuration, Causes of ... 145
Synovitis of the Knee Joint, Cases of, E. B. Weston ... 119

T

Tait, Lawson, Reply to M. Saenger ... 111
Testicle, Cancer of, with Secondary Pleurisy, J. A. Robison ... 114
Tetanus, Bacteria and ... 140
Thyroid, Enlarged, a Cause of Transverse Presentation, Charles Caldwell ... 109
Thompson, J. L., Observations on Displacement of the Crystalline Lens from Congenital and Other Causes ... 85
Tilley, R., Atheroma of the Left Coronary Artery, resulting in Aneurism of the Apex of the Left Ventricle ... 285
Tyrotoxicon in Food ... 139

U

Urine, Retention of ... 157
Uterine Sound, the Chief Source of Danger in the Use of the, G. F. French ... 93

V

Vaginal Hysterectomy, Christian Fenger ... 367
Vagus, Pathology of the Laryngeal ... 173
Virchow, Rudolph, Concerning Myxœdema 30
Visual Troubles, Dyspeptic Origin of ... 156

W

PAGE
Walker, H. O., External Perineal Urethrotomy ........ 94
Weston, Cases of Synovitis of the Knee Joint ........ 119
Wile, W. C., Scirrhus of the Mammæ ...... 80
Will, O. B., Progress in Gynæcology ....... 22
Wing, Elbert, Concerning Myxœdema. Translation ........ 30

### CONTRIBUTORS TO VOLUME LV.

Andrews, Edmund.

Andrews, F. T.

Bombaugh, C. C.

Brown, J. Y.

Davis, N. S., Jr.

Fenger, Christian.

Green, J. E.

Holmes, Bayard.

Martin, F. H.

McGraw, T. A.

Tilley, Robert.

Will, O. B.

### PAMPHLETS RECEIVED.

"The Anatomy and Physiology of the Recurrent Laryngeal Nerves." By Franklin H. Hooper, M. D.

Transactions of the Indiana State Dental Association, 1887.

Transactions of the Illinois State Dental Society, Twenty-third Annual Meeting, 1887.

Medical Communications of the Massachusetts Medical Society, Vol. XIV., No. 1, 1887.

Programme of the Fifteenth Annual Meeting of the American Public Health Association, Nov. 8, 9, 10, 11, 1887.

"Hay Fever." By Seth S. Bishop, M. D.

"A Report on Orthopedic Surgery. Remarks on Stricture of the Rectum." By W. R. Whitehead, M. D.

"On the Existence of Dermatitis Herpetiformis as a Distinct Disease." By Duncan Bulkley, A. M., M. D.

"The Galvanic Treatment of Uterine Fibroids. By Ephraim Cutter, A. M., M. D., LL. D.

"Diet in Cancer." By Ephraim Cutter, A. M., M. D., LL. D.

"To What Extent can we Classify Vesical Calculi for Operation?" By A. Vandeveer, M. D.

"A Case of Gastrotomy for Cancer of the Œsophagus." By J. Collins Warren, M. D.

"Observations on the Cholera Bacillus as a Means of Positive Diagnosis." By S. T. Armstrong, M. D., and J. J. Kinyoun, M. D.

"The True Nature and Definition of Insanity." By C. H. Hughes, M. D.

"An Experimental Study of the Effects of Puncture of the Heart in Chloroform Narcosis." By B. A. Watson, A. M., M. D.

"Supra-Pubic Lithotomy. A Historical Sketch." By Charles W. Dulles, M. D.

"Courier Review Call-Book." Arranged by E. M. Nelson, M. D., Ph. D., J. H. Chambers & Co.

"The Medical News Visiting List." Lea Brothers & Company.

"The Medical World Visiting List."

Transactions of the State Medical Society of Arkansas, 1887.

Transactions of the Medical Society of the State of California, 1887.

Annual Report of the Supervising Surgeon-General of the Marine Hospital Service of the United States, 1887.

Volume LV.] DECEMBER, 1887. [Number 6.

### The CHICAGO MEDICAL JOURNAL AND EXAMINER

EDITOR:

S. J. JONES, M.D., LL.D

COLLABORATORS:

CHICAGO:

PROFESSOR E. ANDREWS, M.D., LL.D.

PROFESSOR J. BARTLETT, M.D.

PROFESSOR W. T. BELFIELD, M.D.

PROFESSOR F. BILLINGS, M.D.

PROFESSOR W. H. BYFORD, A.M., M.D.

PROFESSOR L. CURTIS, A.M., M.D.

PROFESSOR N. S. DAVIS, JR., A.M., M.D.

PROFESSOR O. C. DEWOLF, A.M., M.D.

PROFESSOR E. C. DUDLEY, A.B., M.D.

PROFESSOR C. FENGER, M.D.

PROFESSOR J. N. HYDE, A.M., M.D.

PROFESSOR E. F. INGALS, A.M., M.D.

PROFESSOR W. W. JAGGARD, A.M., M.D.

PROFESSOR F. S. JOHNSON, A.M., M.D.

PROFESSOR H. A. JOHNSON, M.D., LL.D.

PROFESSOR C. W. PURDY, M.D.

PROFESSOR C. G. SMITH, A.M., M.D.

PROFESSOR E. WING, A.M., M.D.

MILWAUKEE, WIS.
PROFESSOR N. SENN, M.D.

WASHINGTON, D. C.:
S. O. RICHEY, M.D.

UNITED STATES ARMY:

SURGEON D. L. HUNTINGTON, M.D.

MARINE HOSPITAL SERVICE:
SURGEON S. T. ARMSTRONG, M.D.

UNITED STATES NAVY:
MEDICAL DIRECTOR J. M. BROWNE, M.D.
MEDICAL DIRECTOR A. L. GIHON, A.M., M.D.

